# Mortality Attributable to Influenza in England and Wales Prior to, during and after the 2009 Pandemic

**DOI:** 10.1371/journal.pone.0079360

**Published:** 2013-12-11

**Authors:** Helen K. Green, Nick Andrews, Douglas Fleming, Maria Zambon, Richard Pebody

**Affiliations:** 1 Respiratory Diseases Department, Centre for Infectious Disease Surveillance and Control, Public Health England, London, United Kingdom; 2 Statistics Department, Centre for Infectious Disease Surveillance and Control, Public Health England, London, United Kingdom; 3 Birmingham Research Unit, Royal College of General Practitioners, Birmingham, United Kingdom; 4 Respiratory Virus Unit, Virus Reference Department, Microbiology Services, Public Health England, London, United Kingdom; Harvard School of Public Health, United States of America

## Abstract

Very different influenza seasons have been observed from 2008/09–2011/12 in England and Wales, with the reported burden varying overall and by age group. The objective of this study was to estimate the impact of influenza on all-cause and cause-specific mortality during this period. Age-specific generalised linear regression models fitted with an identity link were developed, modelling weekly influenza activity through multiplying clinical influenza-like illness consultation rates with proportion of samples positive for influenza A or B. To adjust for confounding factors, a similar activity indicator was calculated for Respiratory Syncytial Virus. Extreme temperature and seasonal trend were controlled for. Following a severe influenza season in 2008/09 in 65+yr olds (estimated excess of 13,058 influenza A all-cause deaths), attributed all-cause mortality was not significant during the 2009 pandemic in this age group and comparatively low levels of influenza A mortality were seen in post-pandemic seasons. The age shift of the burden of seasonal influenza from the elderly to young adults during the pandemic continued into 2010/11; a comparatively larger impact was seen with the same circulating A(H1N1)pdm09 strain, with the burden of influenza A all-cause excess mortality in 15–64 yr olds the largest reported during 2008/09–2011/12 (436 deaths in 15–44 yr olds and 1,274 in 45–64 yr olds). On average, 76% of seasonal influenza A all-age attributable deaths had a cardiovascular or respiratory cause recorded (average of 5,849 influenza A deaths per season), with nearly a quarter reported for other causes (average of 1,770 influenza A deaths per season), highlighting the importance of all-cause as well as cause-specific estimates. No significant influenza B attributable mortality was detected by season, cause or age group. This analysis forms part of the preparatory work to establish a routine mortality monitoring system ahead of introduction of the UK universal childhood seasonal influenza vaccination programme in 2013/14.

## Introduction

Seasonal influenza occurs in annual epidemics in England and Wales, usually peaking around late December/early January. Although the severity and impact of each season depends to some extent on the strain circulating, influenza typically results in mortality each year, the burden of which is predominantly in the elderly and individuals with underlying risk factors for severe influenza. The notable exception in recent years for timing of peaks has been the 2009 pandemic, with the first wave peaking in July 2009 and a second wave peaking in October 2009 [1]. This pandemic strain resulted in mild symptoms in the majority of infected individuals and, in common with previous pandemics [2], the attack rate and mortality predominated in children and young adults.

Quantifying the burden of mortality resulting from influenza is not straightforward. Although mortality data is available in England and Wales with primary cause of death, underreporting of influenza-related deaths is common – either influenza infection is not diagnosed by the clinician or, if influenza is detected, a secondary complication resulting in mortality might be reported rather than the infection. Therefore statistical modelling is needed to indirectly estimate the population-level burden due to influenza and adjust for other factors which temporally coincide with influenza and impact on mortality, including infection with Respiratory Syncytial Virus (RSV) and low temperatures (or cold snaps). Estimates of seasonal influenza burden have been published for various countries using a range of statistical models [3], [4], [5], [6], highlighting both the burden in the elderly and variable activity depending on the strain circulating and the intensity of activity. Global estimates of the mortality burden during the 2009 pandemic have been produced [7], [8] which highlight, along with previous work in England and Wales and other countries [9], [10], [11], [12], the age shift during the pandemic from the elderly towards young adults observed in infection replicated at the level of severe disease, i.e. mortality. Although a decreased mortality burden in terms of absolute number of deaths relative to seasonal influenza was estimated, calculation of potential years of life lost (YLL) which takes account of the younger age distribution during the pandemic has revealed a substantial health burden [3].

While estimation of influenza-attributable mortality prior to and during the 2009 pandemic has previously been calculated in England and Wales, the unusual and varied influenza activity in the post-pandemic seasons suggests different patterns of mortality. In 2010/11, despite apparent widespread infection during the 2009/10 pandemic, the pandemic A(H1N1) strain continued to dominate and circulate in conjunction with influenza B [13]. Influenza activity was reported more often in young and middle aged adults in 2010/11 than in children compared to 2009/10 and was unexpectedly high, with a reported increased impact across various surveillance indicators [13], [14], [15]. Circulation of this virus was not observed in all countries in 2010/11 [16] but even in countries where reported, an increased impact was only observed in a few [17], [18], [19]. In contrast to 2010/11, influenza A(H3N2) was the dominant subtype detected in 2011/12 in England and Wales [20]. Despite low overall seasonal influenza activity, hospitalisations were still reported with the largest proportion in the elderly and outbreaks seen over a number of weeks, particularly in elderly care home settings.

Recommendations have recently been made to extend the routine seasonal influenza vaccination programme to all healthy children aged 2 to 16 years in the UK [21]. Prior to this, there is a need to estimate the attributable burden of mortality due to seasonal influenza through establishing regression methods able to fully disentangle and control for contributing factors during the winter months.

This paper builds on previous work [9] to assess for the first time the relative contribution of influenza A and B to mortality prior to, during and after the 2009 pandemic in England and Wales and, through analysing cause-specific mortality data (determined through coded causes of death), identify the predominant cause-specific groups in which influenza-attributable mortality occurs.

## Materials and Methods

### Data

Weekly data on mortality, respiratory virus activity and temperature were extracted as detailed below and aggregated according to International Organization for Standardization week classification. Analysis was performed for influenza seasons 2006/07 to 2011/12, with each influenza season defined as running from week 40 (beginning of October) to week 20 the following year (mid-May) and an additional season corresponding to the first wave of the pandemic (week 21 2009 to week 39 2009) was reported.

#### Mortality

Individual level mortality data from England and Wales with information on date of death, age and primary cause of death were provided on a weekly basis by the Office for National Statistics. Delays in death registrations are inherent in the system, particularly in young adults [22]. To account for this, data was extracted in February 2013, nine months after the end of the 2011/12 season, by which time more than 90% of deaths during the 2011/12 winter period are reported across all age groups.

The number of deaths were extracted by age group (<15 yrs, 15–44 yrs, 45–64 yrs and 65+yrs) for causes of death previously modelled for determining influenza burden (according to ICD-10 classification [23]): all-cause (all codes), cardiorespiratory causes (I and J codes), respiratory causes (J codes) and pneumonia and influenza (J9–18). Additionally, all-age deaths were extracted by ICD-10 chapter, previously investigated causes likely to be influenced by influenza [4], [24] and causes reported to be a risk factor for severe influenza [25] (Table S1).

#### Viral activity indicators

To indirectly estimate the burden, viral activity each season needs to be accurately represented. Previous work used the number of samples positive for influenza as an indicator of influenza activity [9]. However, differential sampling practice over time can lead to variations between seasons not reflective of true activity. Reporting the proportion of samples positive improves on this but still does not fully capture the severity of influenza strains and impact on the community. Incidence, constructed by combining virological positivity with the community influenza-like illness rate, has recently been used [4], [24], [26], [27]. Weekly all-age data on influenza A, influenza B and RSV positivity (defined as the number of samples positive divided by the number of samples tested for a given virus) was obtained through the RCGP/SMN sentinel general practice swabbing schemes run across England and Wales [20]. No detailed influenza A subtype information was available for the full period of analysis.

All-age RSV positivity may be underrepresented through sentinel swabbing schemes due to a large proportion of samples taken in adults where RSV virus sample tends to be low and less likely to be detected. Therefore to ensure the data was comparable to influenza sampling, the proportion of samples positive were scaled through analysis of RSV positivity from the Respiratory Datamart Scheme (RDS) [1], [13], [20]. This scheme reports positivity from samples received primarily through hospitals, but also community general practices, where RSV testing is likely to be focussed in the paediatric population in whom RSV is more easily detectable. RDS has only been established since the 2009 pandemic, preventing its use in this work for the entire period. Through simple linear regression, the relationship between weekly RSV positivity through the sentinel schemes and weekly RDS RSV positivity was established from 2009 to 2012 and weekly sentinel positivity multiplied up accordingly for the duration of analysis.

Viral incidence values were constructed by multiplying virological positivity with a measure of clinical activity. For influenza, weekly all-age influenza-like illness (ILI) GP consultation rates across England and Wales were used. For RSV, infection typically results in acute bronchitis [28] and so weekly all-age acute bronchitis GP consultation rates across England and Wales were used. Both clinical activity measures were retrieved from the RCGP surveillance scheme [20].

#### Temperature

Daily Central England Temperature (CET) [29] values in degrees Celsius (°C) were collated and weekly averages for mean, minimum and maximum values calculated.

### Model

The structure of age-group-specific generalised linear models presented previously [9] were applied to these new datasets and variables and a similar model selection process undertaken based on Akaike information criterion (AIC) values and observations from surveillance data. A Poisson distribution was used in <15 yrs and 15–44 yr olds and a negative binomial distribution applied when modelling data from 45–64 yr olds and 65+yr olds where overdispersion was detected. All models were fitted with an identity link as the effects of the covariates were assumed to be additive [30]. A full model was fitted to 65+ year olds all-cause mortality and specific terms removed if their exclusion lowered the AIC value. The final model was then applied for other age groups and causes. The initial full model fitted is as follows:
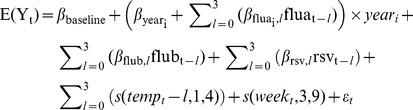
Where:E(Y_t_)  =  estimated number of deaths in week tyear_i_  =  an indicator variable taking the value 1 if year_t_  =  i and 0 otherwise, where i represents each year from i = 1 in 2006/07 to i = 6 in 2011/12flua_i,t_  =  weekly influenza A activity indicator in year i in week t with lags *l* of up to three weeksflub_t_  =  weekly influenza B activity indicator in week t with lags *l* of up to three weeksrsv_t_  =  weekly RSV activity indicator in week t with lags *l* of up to three weekstemp_t_  =  weekly CET measurement in week t with lags *l* of up to three weekss(temp_t_,1,4)  =  b-spline of degree one with knots positioned at 20°C and 27°Cs(week_t_,3,9)  =  cubic b-spline with nine degrees of freedomε_t_  =  error term.

Viral activity indicators were modelled separately as influenza A, influenza B and RSV. Firstly, the form of the viral activity indicator was decided; AIC values were compared when fitting three indicators: the number of positive samples; proportion of samples positive and incidence (positivity*clinical activity) consistently across the viruses. An interaction with year, defined as running from week 20 in one year to week 19 the following year, was included with influenza A to account for the known varying severity of different circulating influenza strains each year. Severity was not assumed to vary by season for influenza B or RSV. A lag of up to three weeks for each virus was included to allow for possible delays from infection to death. Once the indicator had been determined, the significance of removing lag terms for each virus was assessed.

Weekly averages of CET were modelled as a b-spline of degree one with a lag of up to three weeks from temperature to death, allowing for the known delay between a change in temperature and impact on mortality [31]. Firstly, the temperature values modelled, minimum, mean or maximum weekly average CET, were selected through AIC value comparison and then the number of lag terms assessed. The underlying weekly seasonal trend not explained by temperature, influenza and RSV activity was modelled as a cubic b-spline. The number of degrees of freedom was varied and corresponding model AIC values assessed. Finally, knots were modelled in the temperature variable to allow for the non-linear relationship between temperature and mortality and the number and position of the knots varied.

Once finalised, the model was run for each mortality times series and the resulting regression coefficients for each influenza A variable multiplied by the observed values for a given week, summed to give the weekly number of influenza A deaths and subsequently summed across each season. This was replicated for influenza B. 95% confidence intervals (95% CI) were calculated for seasonal estimates of attributable mortality, with the variance composed of the sum of the weekly residual variance (variance of the residuals of the final model) and weekly model prediction variance (square of the standard errors of the prediction of the final model) [9]. If, after summing to get a seasonal estimate, the number of deaths or lower confidence limit was estimated to be less than zero, this was set to zero.

Influenza-attributable mortality rates were also calculated by modelling the number of deaths per 100,000 population by age group. Annual mid-year population estimates were available from ONS [32]. The 2012 estimated population size was not available at the time of analysis and was estimated based on linear regression of previous values. Weekly population estimates were calculated through interpolation of annual estimates and used to determine weekly mortality rates [33].

With an age shift evident during the pandemic, YLL were calculated [3], [34] to take account of the age distribution of deaths. The number of deaths attributable to influenza in each age group each season were multiplied by the life expectancy of that age group, obtained from the World Health Organization [35], and then summed to give an all-age number attributable each season [34].

Mortality data was managed with STATA v12 (StataCorp, College Station, TX) and statistical analyses were carried out using R version 2.15.1 (R Development Core Team, Vienna, Austria).

## Results

### Covariates

**Figure 1:**
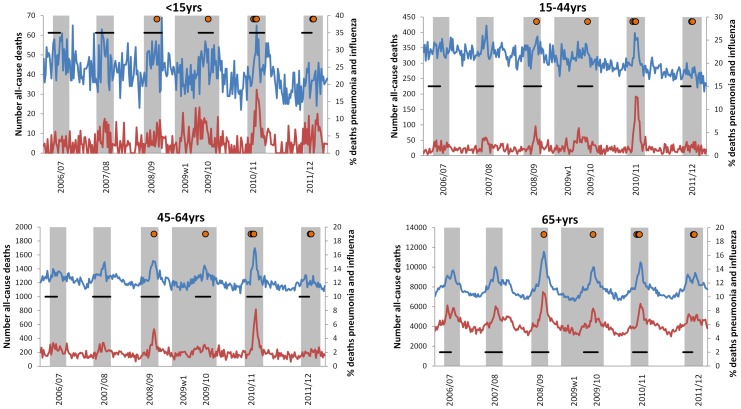
Weekly number of all-cause deaths by age group. The weekly numbers of all-cause deaths are blue and the proportion of those deaths classified as pneumonia and influenza (ICD-10 J9-J18) are red. Weeks shaded grey correspond to significant influenza activity, defined as an influenza incidence proxy (influenza-like illness consultation rates multiplied by proportion of samples positive for influenza) greater than 0 for three consecutive weeks. Horizontal black lines correspond to weeks with significant RSV activity, defined as a RSV incidence proxy (acute bronchitis consultation rates multiplied by proportion of samples positive for RSV) greater than 0 for three consecutive weeks. Orange circles correspond to weeks in which mean CET was below 0°C.

Different patterns in all-cause mortality were observed by age group over the past six years, with clearer peaks seen with increasing age (Figure 1). In 65+ year olds, peaks in mortality were seen each winter with the highest peak in 2008/09, followed by 2010/11. The proportion of deaths coded as pneumonia and influenza corresponded to the peaks in all-cause mortality in terms of relative magnitude and timing. In under 65 year olds, peaks in proportion of pneumonia and influenza deaths only corresponded with all-cause mortality in some seasons, although the largest peak was consistently seen across age groups in 2010/11.

Other mortality causes were analysed, with some showing some fluctuation and winter peaks in mortality (Figures S1 and S2). The number of deaths actually coded as influenza was low, with a peak of 112 in week 1 2011 and a total of 896 (range 19 in 2007/08 to 523 in 2010/11) reported over the six winter seasons. Because of these low numbers, this cause was not analysed, along with other causes because of low numbers reported or inconsistent coding practices across the period of study.

Influenza incidence was temporally consistent with notable peaks in mortality (Figure 1). The highest peaks in influenza A and B activity were reported in 2010/11 when the pandemic A(H1N1) virus continued to circulate (Figure S3). The next highest peaks for influenza A subtypes were seen during the first wave of the pandemic in 2009 (A(H1N1)pdm09) and the winter of 2008/09 (A(H3N2)). Similar peak activity was reported in 2006/07 (A(H3N2)) and 2009/10 (A(H1N1)pdm09), with comparatively lower incidence in 2007/08 (A(H1N1)) and 2011/12 (A(H3N2)) when the lowest peak incidence was reported, which was comparatively late. The peak in incidence was not therefore consistent across the seasons by influenza A subtype. Influenza B additionally circulated in 2007/08 and 2008/09 with lower incidence than seen for influenza A in those seasons. Minimal influenza B activity was observed in 2011/12 and no activity was seen in 2006/07 and 2009/10.

The highest peaks in RSV incidence were seen in 2006/07 and 2009/10 (Figure S3) and a similar peak incidence to influenza A reported in those seasons. Lower peaks in incidence were seen in other seasons with two peaks evident in 2010/11 either side of the peak in influenza activity.

Mean CET followed a cyclical pattern each season inverse to mortality, peaking in June/July and reaching a minimum in December/January, temporally coinciding with peaks in influenza activity and mortality. Cold winters have been seen in the past few years with the lowest mean CET seen in 2010 (−4.2°C in week 51 (2010/11) and −2.6°C in week 1 (2009/10)). Mean weekly temperatures below 0°C were also recorded in 2008/09 (week 1 2009), 2010/11 (weeks 48 and 50) and late in 2011/12 (weeks 5 and 6 2012) (Figure 1).

### Model selection

When assessing representation of viral activity, influenza A, influenza B and RSV incidence (positivity*clinical activity) provided the best model fit compared to the number of positive samples and proportion of samples positive. Including a lag of up to two weeks from infection to death resulted in a better model fit for influenza A and RSV, while the best fit for influenza B was no lag between week of infection and week of death. This discrepancy between the types of influenza is supported by information from surveillance of confirmed influenza fatalities in 2010/11 where A(H1N1)pdm09 confirmed fatalities had a significantly longer lag than B confirmed fatalities (unpublished data). Average weekly mean CET resulted in a better model fit than maximum or minimum CET with a lag of up to two weeks from temperature to death. This delay is consistent with the reported impact of cold weather on mortality [31]. Two knots provided the best fit placed at −3°C and 15°C, corresponding to visible changes in the relationship between the weekly number of deaths and weekly mean CET. The underlying weekly seasonal trend was modelled with 13 degrees of freedom, approximating to a knot every five weeks.

The final model is listed below:
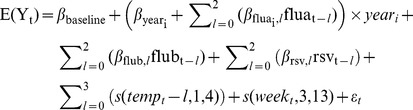
Where:E(Y_t_)  =  estimated number of deaths in week tyear_i_  =  an indicator variable taking the value 1 if year_t_  =  i and 0 otherwise, where i represents each year from i = 1 in 2006/07 to i = 6 in 2011/12flua_i,t_  =  weekly influenza A incidence in week t with lags *l* of up to two weeksflub_t_  =  weekly influenza B incidence in week t with lags *l* of up to two weeksrsv_t_  =  weekly RSV incidence in week t with lags *l* of up to two weekstemp_t_  =  weekly CET measurement in week t with lags *l* of up to two weekss(temp_t_,1,4)  =  b-spline of degree one with knots positioned at −3°C and 15°Cs(week_t_,3,13)  =  cubic b-spline with 13 degrees of freedomε_t_  =  error term.

### Mortality attributable to influenza

**Figure 2:**
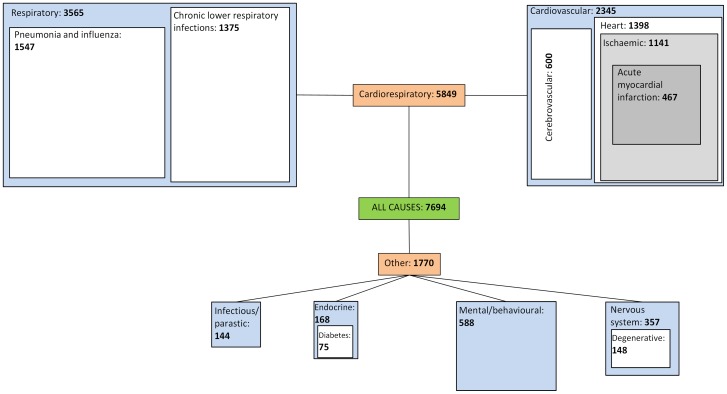
Average influenza A attributable deaths by cause of death. Blue boxes correspond to average all-age significant influenza A attributable deaths by ICD-10 chapter. White and grey boxes correspond to more specific causes of death within ICD-10 chapters and their average all-age significant influenza A attributable deaths. The sizes of the blue, white and grey boxes are proportional to the corresponding number of influenza A deaths attributed. Average values were derived from seasons in which significant influenza attribution was reported overall.

When assessing all-cause, cardiorespiratory, respiratory and pneumonia and influenza mortality, the magnitude of influenza-attributable mortality decreases as the cause becomes more specific. All-age all-cause mortality across England and Wales had a proportion significantly attributed to influenza A in all seasons, except for the two pandemic waves in summer 2009 and 2009/10 (Table 1). The highest peak was seen in 2008/09, with 14,222 (95% CI 11,551–16893) deaths significantly attributed to influenza A, and the lowest in 2007/08 with 4,253 (1,688–6,818) deaths. Significant excess was only seen during the first wave of the pandemic when assessing pneumonia and influenza deaths, with 355 deaths (173–538) attributed. No significant attribution was seen across these causes during the second wave of the pandemic in 2009. An average over seasons either side of the two pandemic waves (2006/07–2008/09 and 2010/11–2011/12) of 7694 all-cause, 5,849 cardiorespiratory, 3,565 respiratory and 1,547 pneumonia and influenza deaths were attributed to influenza (Figure 2). In line with clinical observations, the number of influenza A-attributable deaths and crude rate increased with increasing age group within each season. No significant influenza B-attributable mortality was detected across these causes overall or by age group (Tables 2, 3, 4 and 5).

**Table 1:** Number of influenza A attributable all-cause deaths (95% Confidence Interval) by primary ICD-10 cause of death and influenza season with the dominant circulating influenza A subtype*.

		2006/07	2007/08	2008/09	2009w1 2009/10 2010/11			2011/12
Chapter	Cause	A(H3N2)	A(H1N1)	A(H3N2)	A(H1N1)pdm09			A(H3N2)
I (Infectious/parasitic)		**261 (96–425)**	55 (0–236)	**141 (8–274)**	**89 (23–154)**	86 (0–204)	**320 (160–480)**	28 (0–192)
II (Neoplasms)		588 (0–1316)	499 (0–1325)	**756 (167–1346)**	184 (0–619)	182 (0–905)	4 (0–645)	379 (0–999)
	Malignant neoplasms	585 (0–1301)	417 (0–1210)	**655 (42–1269)**	153 (0–601)	153 (0–861)	3 (0–623)	316 (0–906)
III (Blood)		**55 (4–106)**	33 (0–86)	42 (0–97)	8 (0–38)	6 (0–46)	24 (0–75)	0 (0–46)
IV (Endocrine)		**181 (38–324)**	**166 (20–311)**	**155 (24–287)**	35 (0–119)	35 (0–181)	**219 (80–358)**	**124 (10–239)**
	Diabetes mellitus	90 (0–196)	100 (0–226)	**121 (7–234)**	46 (0–112)	46 (0–168)	**256 (161–350)**	58 (0–175)
V (Mental/behavioural)		6 (0–345)	120 (0–451)	**601 (241–961)**	16 (0–220)	5 (0–387)	**1129 (142–2117)**	**1212 (601–1823)**
VI (Nervous System)		**254 (18–490)**	0 (0–278)	**611 (406–817)**	18 (0–172)	5 (0–288)	**402 (179–624)**	**520 (272–768)**
	Degenerative disorders	124 (0–273)	0 (0–168)	**185 (35–334)**	0 (0–80)	0 (0–191)	**222 (57–386)**	**332 (143–520)**
IX + X (Cardiorespiratory)		**6339 (5065–7614)**	**3584 (1901–5267)**	**11083 (9212–12953)**	460 (0–1370)	462 (0–2107)	**4699 (2804–6595)**	**3541 (2337–4745)**
IX Circulatory		**3153 (2251–4055)**	**1613 (459–2766)**	**3829 (2663–4995)**	56 (0–780)	27 (0–1181)	**1709 (276–3143)**	**1467 (689–2245)**
	Cerebrovascular	**920 (492–1349)**	447 (0–942)	**1211 (644–1778)**	48 (0–387)	24 (0–454)	**867 (296–1438)**	298 (0–648)
	Heart diseases	**2057 (1431–2683)**	**1225 (431–2019)**	**2609 (1898–3319)**	107 (0–631)	45 (0–807)	819 (0–1674)	**1099 (547–1651)**
	Ischaemic	**1331 (888–1774)**	**611 (73–1148)**	**1607 (1103–2110)**	28 (0–422)	7 (0–648)	**2052 (1476–2628)**	**979 (551–1406)**
	AMI^1^	**615 (338–892)**	**480 (208–752)**	**665 (336–995)**	6 (0–164)	3 (0–334)	**366 (135–597)**	**386 (136–635)**
X Respiratory		**3199 (2600–3798)**	**1942 (1187–2698)**	**7213 (6283–8142)**	**469 (110–828)**	471 (0–1232)	**3366 (2633–4099)**	**2105 (1342–2868)**
	Influenza and pneumonia	**1401 (1069–1732)**	**883 (559–1206)**	**3383 (2869–3898)**	**355 (173–538)**	352 (0–747)	**1411 (1035–1788)**	**656 (258–1053)**
	Chronic lower resp. infections	**1211 (904–1518)**	**725 (363–1087)**	**2569 (2162–2977)**	143 (0–345)	129 (0–433)	**1319 (936–1701)**	**1051 (660–1443)**
XI (Digestive system)		236 (0–505)	171 (0–402)	222 (0–522)	104 (0–289)	63 (0–318)	169 (0–423)	163 (0–383)
	Liver disease	80 (0–201)	40 (0–153)	39 (0–160)	90 (0–202)	69 (0–185)	66 (0–206)	43 (0–163)
XIV (Genitourinary system)		203 (0–425)	0 (0–204)	**284 (59–509)**	28 (0–165)	11 (0–207)	70 (0–297)	130 (0–296)
	Renal disease	56 (0–150)	7 (0–104)	50 (0–143)	4 (0–56)	1 (0–90)	51 (0–153)	34 (0–128)
XX (External cause)		14 (0–255)	23 (0–212)	**254 (11–497)**	2 (0–154)	0 (0–234)	24 (0–234)	37 (0–306)
	Traffic	11 (0–111)	27 (0–123)	35 (0–131)	7 (0–67)	2 (0–76)	0 (0–53)	3 (0–60)
**TOTAL (all-cause)**		**8293 (6286–10301)**	**4253 (1688–6818)**	**14222 (11551–16893)**	799 (0–2275)	791 (0–3255)	**5867 (3389–8346)**	**5834 (3539–8129)**

* Bold figures correspond to significant estimates.

1 Acute Myocardial Infarction.

**Table 2:** Number of all- cause influenza-attributable A and B deaths (95% Confidence Interval) and rate per 100,000 population by age group, influenza type and influenza season*.

			2006/07	2007/08	2008/09	2009w1	2009/10	2010/11	2011/12
<15yrs	Influenza A	Number	41 (0–126)	39 (0–136)	15 (0–103)	14 (0–69)	12 (0–85)	40 (0–110)	10 (0–76)
		Rate	0.44 (0.00–1.31)	0.40 (0.00–1.41)	0.15 (0.00–1.06)	0.15 (0.00–0.70)	0.13 (0.00–0.87)	0.40 (0.00–1.10)	0.11 (0.00–0.77)
	Influenza B	Number	0 (0–85)	10 (0–108)	6 (0–94)	0 (0–54)	1 (0–73)	35 (0–104)	2 (0–67)
		Rate	0.00 (0.00–0.88)	0.11 (0.00–1.12)	0.06 (0.00–0.97)	0.00 (0.00–0.56)	0.01 (0.00–0.75)	0.36 (0.00–1.06)	0.02 (0.00–0.68)
15–44yrs	Influenza A	Number	92 (0–317)	**219 (12–426)**	170 (0–421)	**217 (37–397)**	**211 (25–397)**	**436 (246–625)**	39 (0–271)
		Rate	0.39 (0.00–1.39)	**0.97 (0.05–1.88)**	0.76 (0.00–1.87)	**0.96 (0.16–1.75)**	**0.93 (0.11–1.75)**	**1.91 (1.08–2.75)**	0.17 (0.00–1.19)
	Influenza B	Number	1 (0–227)	49 (0–255)	29 (0–280)	0 (0–180)	3 (0–189)	163 (0–352)	8 (0–240)
		Rate	0.01 (0.00–1.01)	0.22 (0.00–1.13)	0.13 (0.00–1.24)	0.00 (0.00–0.80)	0.01 (0.00–0.84)	0.73 (0.00–1.56)	0.04 (0.00–1.06)
45–64yrs	Influenza A	Number	282 (0–772)	**464 (53–875)**	**1004 (459–1548)**	92 (0–456)	92 (0–566)	**1274 (739–1809)**	160 (0–623)
		Rate	2.21 (0.00–5.86)	**3.69 (0.67–6.71)**	**7.47 (3.53–11.41)**	0.70 (0.00–3.32)	0.70 (0.00–4.08)	**8.92 (5.15–12.68)**	1.07 (0.00–4.28)
	Influenza B	Number	0 (0–490)	0 (0–411)	0 (0–545)	0 (0–364)	0 (0–474)	0 (0–535)	0 (0–463)
		Rate	0.00 (0.00–3.65)	0.00 (0.00–3.02)	0.00 (0.00–3.94)	0.00 (0.00–2.61)	0.00 (0.00–3.38)	0.00 (0.00–3.76)	0.00 (0.00–3.21)
65+yrs	Influenza A	Number	**7898 (6130–9666)**	**3565 (1285–5846)**	**13038 (10657–15420)**	499 (0–1750)	485 (0–2584)	**4494 (2191–6798)**	**5846 (3806–7886)**
		Rate	**98.49 (77.98–118.99)**	**48.38 (22.28–74.48)**	**150.57 (123.81–177.33)**	6.92 (0.00–20.87)	6.85 (0.00–30.05)	**47.27 (22.33–72.2)**	**63.32 (41.48–85.16)**
	Influenza B	Number	3 (0–1771)	107 (0–2387)	63 (0–2445)	0 (0–1251)	6 (0–2105)	355 (0–2659)	18 (0–2058)
		Rate	0.07 (0.00–20.57)	2.34 (0.00–28.45)	1.39 (0.00–28.15)	0.00 (0.00–13.94)	0.14 (0.00–23.34)	7.80 (0.00–32.74)	0.39 (0.00–22.23)

* Bold figures correspond to significant estimates.

**Table 3:** Number of cardiorespiratory coded influenza-attributable A and B deaths (95% Confidence Interval) and rate per 100,000 population by age group, influenza type and influenza season*.

			2006/07	2007/08	2008/09	2009w1	2009/10	2010/11	2011/12
<15yrs	Influenza A	Number	10 (0–37)	10 (0–38)	5 (0–27)	8 (0–25)	6 (0–35)	15 (0–44)	20 (0–52)
		Rate	0.11 (0.00–0.38)	0.11 (0.00–0.39)	0.05 (0.00–0.27)	0.08 (0.00–0.25)	0.06 (0.00–0.36)	0.15 (0.00–0.44)	0.21 (0.00–0.53)
	Influenza B	Number	0 (0–27)	2 (0–29)	1 (0–23)	0 (0–17)	0 (0–29)	6 (0–35)	0 (0–32)
		Rate	0.00 (0.00–0.28)	0.02 (0.00–0.30)	0.01 (0.00–0.23)	0.00 (0.00–0.17)	0.00 (0.00–0.30)	0.06 (0.00–0.35)	0.00 (0.00–0.33)
15–44yrs	Influenza A	Number	54 (0–121)	**165 (86–245)**	**141 (48–233)**	**87 (14–160)**	85 (0–182)	**282 (185–379)**	51 (0–137)
		Rate	0.24 (0.00–0.53)	**0.73 (0.38–1.08)**	**0.62 (0.21–1.03)**	**0.38 (0.06–0.71)**	0.37 (0.00–0.80)	**1.24 (0.82–1.67)**	0.22 (0.00–0.60)
	Influenza B	Number	0 (0–68)	6 (0–86)	4 (0–96)	0 (0–73)	0 (0–97)	20 (0–117)	1 (0–86)
		Rate	0.00 (0.00–0.30)	0.03 (0.00–0.38)	0.02 (0.00–0.43)	0.00 (0.00–0.32)	0.00 (0.00–0.43)	0.09 (0.00–0.52)	0.00 (0.00–0.38)
45–64yrs	Influenza A	Number	**356 (103–610)**	**414 (191–638)**	**737 (417–1057)**	101 (0–271)	101 (0–361)	**1052 (725–1379)**	159 (0–373)
		Rate	**2.73 (0.84–4.62)**	**3.22 (1.58–4.86)**	**5.44 (3.12–7.75)**	0.75 (0.00–1.96)	0.74 (0.00–2.60)	**7.29 (4.99–9.59)**	1.08 (0.00–2.56)
	Influenza B	Number	0 (0–254)	8 (0–231)	4 (0–325)	0 (0–169)	0 (0–261)	25 (0–352)	1 (0–215)
		Rate	0.00 (0.00–1.89)	0.09 (0.00–1.73)	0.05 (0.00–2.37)	0.00 (0.00–1.22)	0.01 (0.00–1.86)	0.29 (0.00–2.59)	0.01 (0.00–1.49)
65+yrs	Influenza A	Number	**5934 (4803–7065)**	**2997 (1407–4587)**	**10179 (8523–11835)**	281 (0–1084)	281 (0–1757)	**3538 (1824–5253)**	**3378 (2240–4515)**
		Rate	**72.69 (59.57–85.81)**	**39.62 (21.43–57.82)**	**116.92 (98.30–135.54)**	3.99 (0.00–12.94)	4.00 (0.00–20.32)	**37.31 (18.75–55.88)**	**36.08 (23.91–48.26)**
	Influenza B	Number	2 (0–1134)	83 (0–1673)	49 (0–1705)	0 (0–803)	5 (0–1481)	276 (0–1991)	14 (0–1151)
		Rate	0.05 (0.00–13.17)	1.66 (0.00–19.85)	0.98 (0.00–19.60)	0.00 (0.00–8.95)	0.10 (0.00–16.41)	5.51 (0.00–24.08)	0.27 (0.00–12.45)

* Bold figures correspond to significant estimates.

**Table 4:** Number of respiratory coded influenza-attributable A and B deaths (95% Confidence Interval) and rate per 100,000 population by age group, influenza type and influenza season*.

			2006/07	2007/08	2008/09	2009w1	2009/10	2010/11	2011/12
<15yrs	Influenza A	Number	7 (0–25)	2 (0–22)	1 (0–18)	8 (0–20)	7 (0–30)	20 (0–46)	19 (0–45)
		Rate	0.07 (0.00–1.12)	0.02 (0.00–1.07)	0.01 (0.00–1.02)	0.09 (0.00–0.76)	0.07 (0.00–0.97)	0.20 (0.00–1.87)	0.19 (0.00–1.21)
	Influenza B	Number	0 (0–18)	0 (0–20)	0 (0–18)	0 (0–11)	0 (0–24)	0 (0–26)	0 (0–26)
		Rate	0.00 (0.00–0.19)	0.00 (0.00–0.21)	0.00 (0.00–0.18)	0.00 (0.00–0.11)	0.00 (0.00–0.24)	0.00 (0.00–0.26)	0.00 (0.00–0.26)
15–44yrs	Influenza A	Number	**43 (7–79)**	**81 (44–117)**	**100 (66–135)**	**67 (36–99)**	**66 (20–113)**	**214 (160–268)**	15 (0–48)
		Rate	**0.19 (0.03–0.35)**	**0.36 (0.20–0.52)**	**0.44 (0.29–0.60)**	**0.30 (0.16–0.44)**	**0.29 (0.09–0.50)**	**0.94 (0.70–1.18)**	0.07 (0.00–0.21)
	Influenza B	Number	0 (0–36)	1 (0–37)	0 (0–35)	0 (0–32)	0 (0–47)	3 (0–57)	0 (0–32)
		Rate	0.00 (0.00–0.16)	0.00 (0.00–0.17)	0.00 (0.00–0.15)	0.00 (0.00–0.14)	0.00 (0.00–0.21)	0.01 (0.00–0.25)	0.00 (0.00–0.14)
45–64yrs	Influenza A	Number	**174 (40–309)**	**156 (34–279)**	**530 (388–672)**	**96 (19–174)**	96 (0–221)	**786 (640–933)**	83 (0–179)
		Rate	**1.35 (0.35–2.36)**	**1.25 (0.35–2.15)**	**3.91 (2.89–4.94)**	**0.70 (0.14–1.25)**	0.70 (0.00–1.58)	**5.53 (4.49–6.56)**	0.55 (0.00–1.21)
	Influenza B	Number	0 (0–134)	0 (0–123)	0 (0–142)	0 (0–77)	0 (0–124)	0 (0–146)	0 (0–96)
		Rate	0.00 (0.00–1.00)	0.00 (0.00–0.90)	0.00 (0.00–1.03)	0.00 (0.00–0.55)	0.00 (0.00–0.89)	0.00 (0.00–1.03)	0.00 (0.00–0.66)
65+yrs	Influenza A	Number	**2976 (2419–3532)**	**1719 (986–2452)**	**6584 (5721–7447)**	323 (0–665)	322 (0–1035)	**2377 (1688–3066)**	**2026 (1280–2773)**
		Rate	**36.48 (30.03–42.94)**	**22.81 (14.43–31.19)**	**75.35 (65.65–85.06)**	**4.03 (0.22–7.85)**	4.04 (0.00–11.92)	**25.03 (17.56–32.49)**	**21.02 (13.03–29.00)**
	Influenza B	Number	1 (0–558)	39 (0–772)	23 (0–887)	0 (0–343)	2 (0–715)	131 (0–820)	7 (0–753)
		Rate	0.02 (0.00–6.47)	0.76 (0.00–9.14)	0.45 (0.00–10.16)	0.00 (0.00–3.82)	0.05 (0.00–7.92)	2.54 (0.00–10.00)	0.13 (0.00–8.11)

* Bold figures correspond to significant estimates.

**Table 5:** Number of pneumonia and influenza coded influenza-attributable A and B deaths (95% Confidence Interval) and rate per 100,000 population by age group, influenza type and influenza season*.

			2006/07	2007/08	2008/09	2009w1	2009/10	2010/11	2011/12
<15yrs	Influenza A	Number	8 (0–19)	1 (0–16)	2 (0–12)	8 (0–17)	7 (0–22)	**20 (7–33)**	**15 (2–27)**
		Rate	0.08 (0.00–0.19)	0.02 (0.00–0.16)	0.02 (0.00–0.12)	0.08 (0.00–0.18)	0.07 (0.00–0.22)	**0.20 (0.07–0.33)**	**0.15 (0.02–0.27)**
	Influenza B	Number	0 (0–11)	0 (0–14)	0 (0–10)	0 (0–9)	0 (0–15)	0 (0–13)	0 (0–12)
		Rate	0.00 (0.00–0.11)	0.00 (0.00–0.15)	0.00 (0.00–0.11)	0.00 (0.00–0.09)	0.00 (0.00–0.15)	0.00 (0.00–0.13)	0.00 (0.00–0.13)
15–44yrs	Influenza A	Number	10 (0–36)	**40 (13–66)**	**53 (26–80)**	**55 (26–83)**	**54 (15–94)**	**171 (132–210)**	4 (0–28)
		Rate	0.05 (0.00–0.16)	**0.18 (0.06–0.29)**	**0.23 (0.11–0.35)**	**0.24 (0.12–0.37)**	**0.24 (0.07–0.41)**	**0.75 (0.58–0.93)**	0.02 (0.00–0.12)
	Influenza B	Number	0 (0–26)	7 (0–33)	4 (0–31)	0 (0–28)	0 (0–40)	23 (0–62)	1 (0–25)
		Rate	0.00 (0.00–0.12)	0.03 (0.00–0.15)	0.02 (0.00–0.14)	0.00 (0.00–0.13)	0.00 (0.00–0.18)	0.10 (0.00–0.27)	0.01 (0.00–0.11)
45–64yrs	Influenza A	Number	57 (0–123)	**117 (68–166)**	**251 (187–314)**	**60 (20–101)**	59 (0–131)	**364 (294–435)**	35 (0–88)
		Rate	0.44 (0.00–0.94)	**0.88 (0.52–1.25)**	**1.83 (1.37–2.29)**	**0.43 (0.14–0.72)**	0.43 (0.00–0.94)	**2.55 (2.05–3.05)**	0.23 (0.00–0.60)
	Influenza B	Number	0 (0–66)	9 (0–58)	5 (0–69)	0 (0–40)	1 (0–73)	30 (0–100)	1 (0–54)
		Rate	0.00 (0.00–0.49)	0.07 (0.00–0.43)	0.04 (0.00–0.50)	0.00 (0.00–0.29)	0.00 (0.00–0.52)	0.24 (0.00–0.74)	0.01 (0.00–0.38)
65+yrs	Influenza A	Number	**1325 (1010–1641)**	**746 (427–1065)**	**3097 (2587–3607)**	**256 (76–437)**	254 (0–626)	**895 (536–1254)**	**613 (223–1003)**
		Rate	**16.27 (12.62–19.93)**	**9.75 (6.10–13.40)**	**35 15 (29.42–40.89)**	**3.02 (1.01–5.04)**	3.00 (0.00–7.10)	**9.17(5.28–13.06)**	**6.25 (2.08–10.42)**
	Influenza B	Number	2 (0–317)	54 (0–374)	32 (0–542)	0 (0–181)	3 (0–375)	181 (0–541)	9 (0–399)
		Rate	0.02 (0.00–3.68)	0.76 (0.00–4.41)	0.45 (0.00–6.18)	0.00 (0.00–2.02)	0.04 (0.00–4.15)	2.51 (0.00–6.40)	0.13 (0.00–4.30)

* Bold figures correspond to significant estimates.

Prior to the 2009 pandemic, the burden of influenza-attributed mortality was predominantly in 45+ year olds with the highest impact seen in 2008/09 when A(H3N2) circulated. The burden of 2008/9 in 65+ year olds was evident with the highest attributed mortality across the six year times series: 13,038 (10,657–15,420) all-cause deaths, equating to a crude rate of 150.6 per 100,000 population. No significant attributable mortality was seen in under 15 year olds in the pre-pandemic period. The largest number attributable in 15–44 year olds was seen in 2008/09 and 2007/08, with little detected in 2006/07.

Conversely during the pandemic, influenza-attributable mortality was focussed in 15–44 year olds, the only age group in which significant influenza A attributable mortality was detected during the second wave of the pandemic with 211 (25–397) all-cause deaths and 54 (15–94) pneumonia and influenza deaths attributed. During the first wave of the pandemic, significant excess all-cause mortality was only seen in this age group, with excess only significant in older age groups when looking at respiratory deaths. For pneumonia and influenza deaths, 55 (26–83) respiratory deaths were attributed in 15–44 year olds, 60 (20–101) in 45–64 year olds and 256 (76–437) in 65+ year olds.

**Figure 3:**
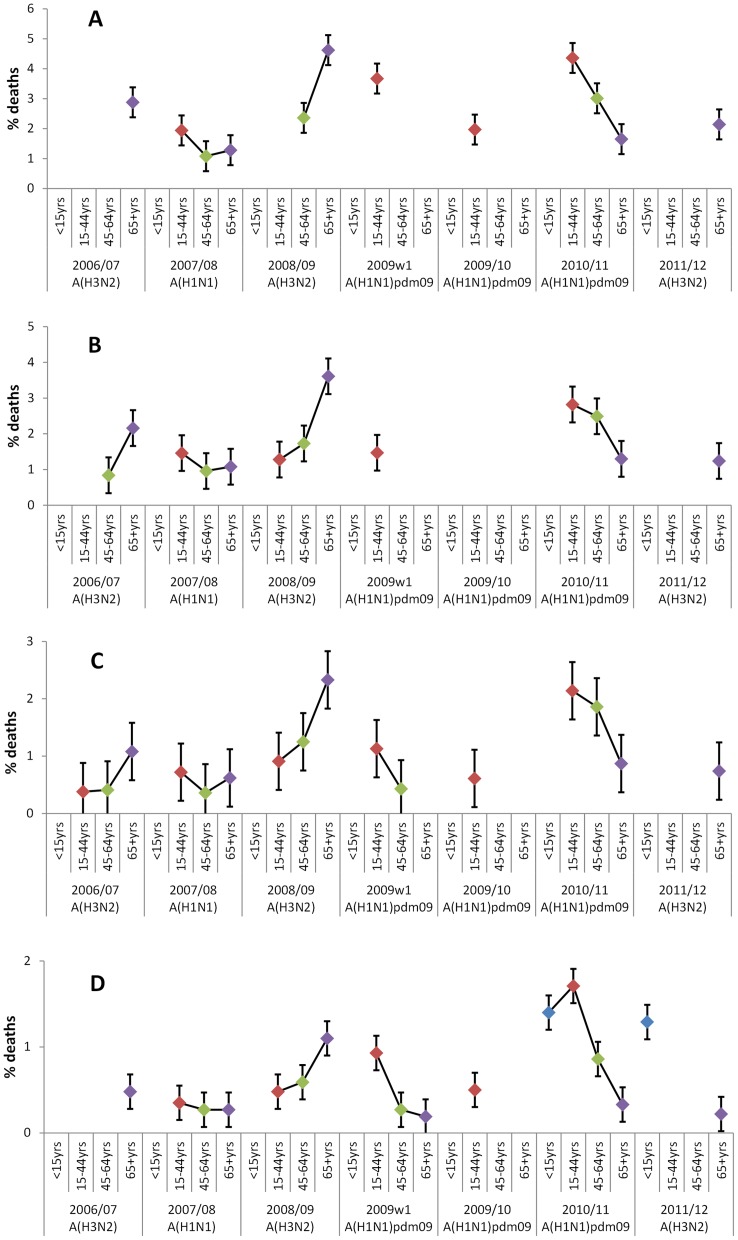
Proportion of all-cause deaths attributable to influenza A by age group and season when assessing coded causes of death. Proportion of all-cause deaths attributable to influenza A by age group and season where significant (corresponding confidence intervals not crossing 0). The number of influenza A-attributable deaths correspond to the following datasets analysed: all-cause deaths (Figure 3a), cardiorespiratory deaths (Figure 3b), respiratory deaths (Figure 3c) and pneumonia and influenza deaths (Figure 3d). Dominant influenza A subtype by season is indicated.

In the first post-pandemic season in 2010/11 when A(H1N1)pdm09 continued to circulate, the start of an age shift in mortality from the observations in 2009/10 was seen, although increased burden in younger age-groups persisted (Figure 3). The highest number of attributable deaths and crude rates were seen across causes (all-cause (Figure 3a), cardiorespiratory (Figure 3b), respiratory (Figure 3c) and pneumonia and influenza deaths (Figure 3d)) in under 65 year olds compared to other seasons analysed, with the proportion of all-cause deaths attributable to influenza in 15–44 year olds (4.4% of deaths in 15–44 year olds in 2010/11) similar to the proportion of deaths in 65+ year olds in 2008/09 attributable to influenza (4.6%). 895 (536–1,254) pneumonia and influenza deaths were attributed in 65+ year olds, equating to a proportion of 0.3% of all-cause deaths, less than seen in under 65 year olds (1.7% in 15–44 year olds, 1.4% in <15 year olds and 1.0% in 45–64 year olds).

In 2011/12 when A(H3N2) was circulating, there was no significant influenza-attributable mortality detected in 15–44 and 45–64 year olds in all-cause, cardiorespiratory, respiratory or pneumonia and influenza deaths. Significant excess was seen in 65+ year olds, with all-cause attribution (5,834 deaths, 3,539–8,346) similar to that seen in 2010/11 (5,867 deaths, 3,389–8,346). However as the cause became more specific, the relative proportion of influenza-attributable deaths compared to 2010/11 decreased and became more similar to low numbers seen in 2007/08 when A(H1N1) was circulating. Significant excess was also seen in under 15 year olds in pneumonia and influenza coded deaths (15, 2–27).

YLL quantification estimated a seasonal average of 55,238 YLL attributed to influenza A strains other than the pandemic strain with all-cause data. The highest number in non-pandemic years was seen in 2006/07 (84,907 YLL). During the two waves of the pandemic in 2009/10, a total of 39,032 YLL were attributed with all-cause data, 71% of the average number attributable to other A subtypes. The largest YLL burden was seen in 2010/11 when A(H1N1)pdm09 continued to circulate (105,013 YLL). Incorporating with the above 2009/10 pandemic estimates attributed a total of 144,045 YLL to that strain for the period from 2009 to 2011. With pneumonia and influenza mortality data, a seasonal average of 15,970 YLL were attributed to influenza A strains other than the pandemic strain, with the highest number seen in 2008/09 (37,781 YLL). 15,035 YLL were attributed to A(H1N1)pdm09 during the two waves of the pandemic in 2009 with pneumonia and influenza mortality data, 94% of the average seasonal number. The total attributable to that strain was 44,689 YLL when 2010/11 was included (29,654 YLL).

When analysing the contribution of various causes to average all-cause influenza A attribution through independently fitted regression models, an additive relationship was observed (Table 1 and Figure 2). The proportion of all-cause attributable influenza deaths coded as cardiorespiratory in seasons either side of the two pandemic waves averaged 76%, although the proportion in 2011/12 was less than seen in other seasons (61% vs. 76–84%). With further cause-specific analysis, approximately 60% were attributable to deaths coded as respiratory and 40% to cardiovascular causes. Within respiratory deaths, an average of 43% was assigned to pneumonia and influenza coded deaths and 67% of the other respiratory causes attributed to lower respiratory infections. Within cardiovascular deaths, the majority (66%) were found in heart causes.

The remaining 24% not attributed to cardiorespiratory deaths were detected in other causes. Significance was reached by ICD-10 chapter in certain infectious and parasitic causes, endocrine causes (within which 44.6% were attributed to diabetes), mental and behavioural disorders and nervous system causes (within which 41.5% were attributed to degenerative nervous system). There was still no significant attributable excess during the two waves of the pandemic in causes other than cardiorespiratory apart from certain infectious and parasitic causes in the first wave (141 (8–294) deaths). Deaths resulting from traffic accidents were included as a control – no significant influenza-attributable excess was detected overall or by season for this group.

## Discussion

Influenza-attributable mortality has varied by season over the past few years and in particular by age group. Following a severe influenza A(H3N2) season in 2008/09 in 65+ year olds, attributed mortality was low during the A(H1N1) pandemic in this age group, with no significant influenza-attributable mortality detected during the winter of 2009/10 and comparatively low levels seen the following seasons. This is in contrast with 15–64 year olds where the largest burden in the past six seasons was seen in 2010/11 due to A(H1N1)pdm09, additionally highlighted by the YLL attributed during this season. No significant excess was seen in <15 year olds prior to 2010/11. The pattern of mortality during the 2009/10 pandemic by age group mirrors that of earlier pandemics with a disproportionate effect on younger adults. When all-age influenza excess mortality was assessed by cause of death, the majority was attributed to respiratory and cardiovascular causes but nearly a quarter was detected in other causes. For all age groups, no significant influenza B attributable mortality was detected by season or cause.

Prior to the pandemic, the highest overall burden was seen in 2008/09 in 65+ year olds when A(H3N2) was circulating. The severity of this strain in this season in the elderly in the UK has been reported previously through various surveillance data sources [36]. Conversely, A(H1N1) circulation in 2007/08 resulted in a comparatively larger impact in 15–44 yrs and a smaller one in 65+ year olds. This is similar to other work outside the UK which attributes a higher burden of influenza-associated mortality (as measured by excess mortality rate) to A(H3N2) relative to A(H1N1) [4], [37]. For comparable seasons and age groups, the all-cause estimates are lower than previously reported in the UK [9], [38], although these analyses were done on earlier seasons of data with higher levels of influenza activity. Additionally, previous work looked at positive counts of respiratory virus [9], rather than considering the proportion of samples positive and clinical activity, which could overestimate attributable mortality [26]. Sensitivity analysis with the model confirmed this with an average of 9,886 all-age all-cause deaths (7,603–12,169) attributed with positive counts, compared to 7,333 (4,624–10,042) using positivity and 7,628 (5,219–10,037) using incidence. Nevertheless, influenza attribution is fairly robust to the model fitted with various indicators of influenza and, as reported elsewhere [4], [24], the use of a combination indicator incorporating virological and clinical observations gave a good model fit and reflected influenza activity observed in the community.

Crude all-age rates in these three pre-2009 pandemic seasons average 16.3 per 100,000 all-cause deaths to 3.5 per 100,000 pneumonia and influenza, driven largely by the severity of 2008/09. Other pre-pandemic figures produced globally in the US [24], Hong Kong [4], Spain [5] and Brazil [6] report a comparable lower level of around 11 per 100,000 all-cause deaths and 1–2 per 100,000 pneumonia and influenza deaths. However these analyses were done pre-2009 pandemic and looked at a larger number of seasons back to the early 1990s. Additionally, during the 2008/09 season, a less severe A(H1N1) strain predominated in the Americas [39] compared to A(H3N2) in Europe.

During the 2009 pandemic, little excess influenza mortality was seen [9]. Overall, 1,590 all-cause deaths during the first two waves in 2009/10 were attributed to the pandemic strain with a near equal distribution between the two waves, although neither wave reached significance. Crude rates averaged 1.4 per 100,000 all-cause deaths and 0.6 per 100,000 pneumonia and influenza deaths which is less than the pre-pandemic estimates above. 15–44 year olds were most affected and excess was only detected in older age groups when more specific respiratory cause data was analysed. In previous work, no significant attribution was detected in any age group during the two waves and the estimates here are higher for comparable age groups [9]. Countries in the northern hemisphere who have published estimates of influenza-attributable mortality during the pandemic [10], [11], [12], [37] and global estimates [7], [8] confirm the pattern seen here, with a lower burden compared to estimated typical seasonal influenza and the burden primarily localised in young people. Compared to the reported A(H1N1)pdm09 confirmed fatalities in England during the pandemic, the estimated all-age number is approximately three times higher (1,590 vs. 492). The distribution of attributed deaths differed to confirmed reports with the majority of confirmed cases occurring during the second wave and a higher proportion in younger age groups, although significance in this model was primarily seen for 15–44 year olds (not in the 65 plus age-group).

In the first post-pandemic season in 2010/11, 15–44 year olds were notably affected, with the largest excess reported in seasons analysed here and previously [9]. Attributable mortality was over double seen in previous seasons in all-cause deaths and over triple in pneumonia and influenza deaths, with YLL calculation highlighted the burden further. The burden in 45–64 year olds was also high, with this age group notably affected both in this season and in 2008/09. Significant attributable mortality was also detected in 65+ year olds in 2010/11 (whereas it was not in 2009/10), though not to the extent of under 65 year olds. While the relative increase in impact of the pandemic strain from 2009/10 to 2010/11 has been reported elsewhere for both influenza confirmed hospital and intensive care admissions and fatalities in England and Wales [13], this is the first time it has been demonstrated through population level mortality attribution.

2011/12 was considered to be a comparatively mild influenza season with the sparing of significant attributable mortality in 15–64 year olds. However, significant excess was observed in the elderly, consistent with numerous reports of outbreaks of influenza A(H3N2) in vulnerable institutions such as care homes, often with associated fatalities [20]. Cause-specific analysis attributed proportionally fewer influenza deaths to cardiorespiratory causes, with the figure more similar to that seen in the 2007/08 A(H1N1) season than previous A(H3N2) seasons. Therefore despite some surveillance schemes suggesting low influenza activity, it is important not to underestimate the impact of influenza, particularly A(H3N2) on mortality.

Crude rates in these two post-pandemic seasons average 10.4 per 100,000 all-cause deaths and 1.9 per 100,000 pneumonia and influenza, more than during the pandemic but less than seen pre-pandemic which is likely a result of burden in young people in 2010/11 (rather than the elderly) and a mild influenza season in 2011/12. Estimates of burden during these seasons from other countries have yet to be published and it will be interesting to compare observations once estimates are available. In contrast with the UK, influenza activity in 2011/12 generally had higher intensity levels compared to 2010/11 in several European countries [40] and in 2012/13, other countries have reported severe activity associated with A(H1N1)pdm09 and A(H3N2) [41], [42]. The considerable variation by age group and season of influenza-attributable mortality in recent years highlights the importance of regular seasonal analysis of excess mortality and providing age-group specific estimates, in particular when interpreting the impact of interventions such as vaccination.

No significant influenza B attributable mortality was detected by season, age group or cause. Low numbers were detected apart from in 2010/11 where 4% of all-cause influenza-attributable deaths resulted from influenza B, a similar proportion to that seen in confirmed fatalities across the UK (7%, 40/582) [13]. Significance may not have been reached due to the sensitivity of the model. However the low numbers attributed suggest the contribution of influenza B to influenza mortality in England and Wales is low as seen in other countries [4], [24]. Analysis of the 2012/13 season in England and Wales when data is available will be useful to compare, with the season initiated by circulation of influenza B followed later by influenza A(H3N2) and peaks in excess all-cause mortality temporally coinciding with circulation of both types [43], [44].

This is the first time cause-specific influenza-attributable mortality modelling has been done in England and Wales since the 2009 pandemic. The majority of influenza deaths were detected in respiratory and cardiovascular causes, the proportion of which was similar to recent estimates elsewhere [4], [24] with a low number of deaths actually coded as influenza each season. During the pandemic, excess mortality attributable to influenza only reached significance in certain age groups when assessing pneumonia and influenza mortality data despite infection-confirmed deaths reported in those age groups. Modelling of cardiorespiratory cause data provides increased specificity for influenza-attributable mortality, however nearly a quarter of influenza-attributable deaths were detected in other causes. Therefore it is important to undertake estimates using all-cause data to provide an upper estimate, with increased specificity provided when using cardiorespiratory and respiratory end-points.

In causes other than cardiorespiratory, significant attribution was seen in ICD-10 groups such as mental and behavioural disorders (Chapter V), nervous system (including degenerative nervous system conditions) (Chapter VI), endocrine (including diabetes) (Chapter IV) and certain infectious and parasitic diseases (Chapter I). Statistically estimated influenza mortality attributed to deaths coded as resulting from mental and behavioural disorders has not previously been investigated in depth. However influenza outbreaks are reported in dementia care units [45] and looking at the time series there is a clear seasonality, with peaks in winter months and an increasing trend in recent seasons. Infectious and parasitic disease deaths also had winter peak seasonality and significant attribution in some seasons. One potential explanation is the coding of secondary bacterial infection, following influenza infection, as primary cause of death. More detailed assessment of codes within these chapters resulting in peaks could determine the pathogens implicated and if attribution to influenza is likely.

Overall when comparing cause attribution with other countries, the proportion attributed to degenerative nervous system conditions and diabetes was similar to the US [24]. There was no significant average attribution to neoplasm, digestive, genitourinary and external cause deaths, which persisted when specific causes within the chapters were assessed (malignant neoplasms, chronic liver conditions, chronic renal conditions and traffic-associated deaths respectively). There was no significant attribution to renal disease, despite results similar to pre-pandemic observations in the US [24] and Hong Kong [4] and it being known to be a risk factor for severe influenza [25]. Aside from model sensitivity, further work needs to look at procedures underlying coding this condition as a primary mortality cause to help determine if lack of significant attribution is true. For all causes, despite a standard ICD10 coding system between countries, it is difficult to be confident in comparability as coding procedures are likely to differ; evaluation of these practices between countries is recommended [34].

It is important to have different statistical approaches to estimating influenza mortality due to underreporting as a cause of death. Further work should be done on assessing different statistical models for attributing mortality and to have results as comparable as possible between countries [46]. As seen previously, the model fits and better predicts when peaks in mortality are evident in older age groups (Figures S4, S5, S6 and S7). Despite known influenza fatalities occurring in <15 year olds, little significant influenza-attributable mortality was detected, with some influenza A attributed pneumonia and influenza deaths in 2010/11 and 2011/12. While this suggests that influenza has little impact on mortality in under 15 year olds, the model had a better fit in older age groups and did not fully capture spikes in younger age groups, suggesting this type of modelling is unsuitable for detection in mortality patterns without clear peaks [9], even with cause-specific data. Assessing impact on mortality in this age group might therefore be more effective by another approach such as individual level active surveillance for fatal influenza-related deaths or proxy indicators such as proportion of death registrations with specific cause of death codes. Use of virological and clinical data by age group may better capture these peaks, although small sample sizes by age group may result in indicators that are too noisy. Further work is required to assess the pattern of age-specific indicators of influenza activity and how much this impacts on calculated mortality attribution.

RSV activity and temperature were controlled for in this analysis. In the previous manuscript [9], the number of positive samples was modelled and RSV counts were very high in comparison to influenza counts which may have resulted in an overestimation of RSV-attributable mortality. However underestimation may have resulted through calculation of incidence, with comparatively lower values and less clear peaks defined. This may have resulted from insufficient scaling of the number of samples positive through sentinel swabbing schemes. Due to the potential biases of RSV sampling and detection by age group, as mentioned above the use of age-specific virological data rather than an all-age total may better capture virological activity. Additionally, the use of all-age acute bronchitis as a clinical indicator may not have been specific enough to RSV and influenced by other viruses [28]. Work needs to be done to assess if age-specific acute bronchitis rates provide added benefit or if there is another more sensitive clinical measure of RSV activity to define the most suitable indicator of RSV activity comparable to influenza. Sensitivity analyses looking at the impact of RSV on influenza estimates showed little variation in influenza estimates when RSV was excluded from the model.

While RSV typically circulates prior to influenza, making its effects easier to separate, low temperatures coincided with influenza activity each winter, even when the latter was comparatively late in 2011/12, perhaps suggesting an interaction. It is therefore difficult to separate the effects of these two variables; further work should be done on understanding and quantifying the interplay between them. The model attributes a considerable amount of unaccounted seasonally varying mortality to temperature – further work is thus required to determine what other additional factors not considered here such as other viruses or bacteria or climatic dynamics might contribute to this variation.

When interpreting this work, it is important to consider that this is an ecological analysis. Additionally, region-specific variations have not been assessed in this paper; a distinctive geographic pattern of influenza transmission occurred in England during the pandemic [1] and it would be interesting to quantify the burden by region adjusting for underlying risk differences. When assessing the data, only all-age values were used to calculate activity indicators, preventing age-group specific values being constructed which may more accurately capture age group specific attribution. Influenza A subtype specific attributable mortality was based on the dominant circulating strain each season as detailed subtype information was not available for the full period of analysis. Once sufficient data is available, influenza A subtypes can be modelled separately. For cause-specific analysis, only primary underlying cause of death statistic was available which may result in either underestimation or overestimation of the number of deaths reported by cause and subsequently the number of deaths attributable to influenza. When calculating YLL, the impact of an individual being in a risk group for severe influenza on their life expectancy independent of influenza infection was not considered. The proportion of fatal influenza cases in a clinical risk group are typically higher than seen in the general population due to increased likelihood of a severe outcome. Not accounting for this will overestimate the burden of YLL attributed to influenza. Further work needs to assess and quantify this.

In conclusion, influenza-attributable mortality varied considerably by season and by age group, highlighting the importance of regular seasonal analysis. In concordance with other infection and severity indicators, the burden of seasonal influenza shifted from the elderly to young adults when the 2009 pandemic strain was circulating which continued into 2010/11 when a larger impact was seen. In 2011/12 significant excess was still again seen in the elderly despite an overall mild season, although impact was not as severe as in previous A(H3N2) seasons. While the majority of influenza-attributable mortality was detected in cardiorespiratory deaths, it was also seen in other causes, highlighting the importance of continuing to produce all-cause estimates as well as cause-specific ones.

## Supporting Information

Figure S1 **Observed number of all-age weekly deaths by primary cause of death (blue) and** **expected deaths from final model (red) for cardiorespiratory causes.**
(TIF)

Figure S2 **Observed number of all-age weekly deaths by primary cause of death (blue) and** **expected deaths from final model (red) for causes other than cardiorespiratory.**
(TIF)

Figure S3 **Weekly incidence proxy (clinical activity multiplied by proportion of samples positive) for influenza A, influenza B and respiratory syncytial virus (RSV) and weekly means of minimum, mean and maximum Central England Temperature (CET), 2006–2012.**
(TIF)

Figure S4 **Observed number of weekly deaths by age group (blue) and expected deaths from** **final age-specific models (red) for all-cause deaths.**
(TIF)

Figure S5 **Observed number of weekly deaths by age group (blue) and expected deaths from** **final age-specific models (red) for cardiorespiratory deaths.**
(TIF)

Figure S6 **Observed number of weekly deaths by age group (blue) and expected deaths from** **final age-specific models (red) for respiratory deaths.**
(TIF)

Figure S7 **Observed number of weekly deaths by age group (blue) and expected deaths from** **final age-specific models (red) for pneumonia and influenza deaths.**
(TIF)

Table S1 **List of ICD-10 codes extracted with description.**
(DOCX)
