# Supplementary figures and images for: Mortality Attributable to Influenza in England and Wales Prior to, during and after the 2009 Pandemic
Source: PLoS One. 2013 Dec 11;8(12):e79360. doi: 10.1371/journal.pone.0079360 (PMC3859479; doi:10.1371/journal.pone.0079360)

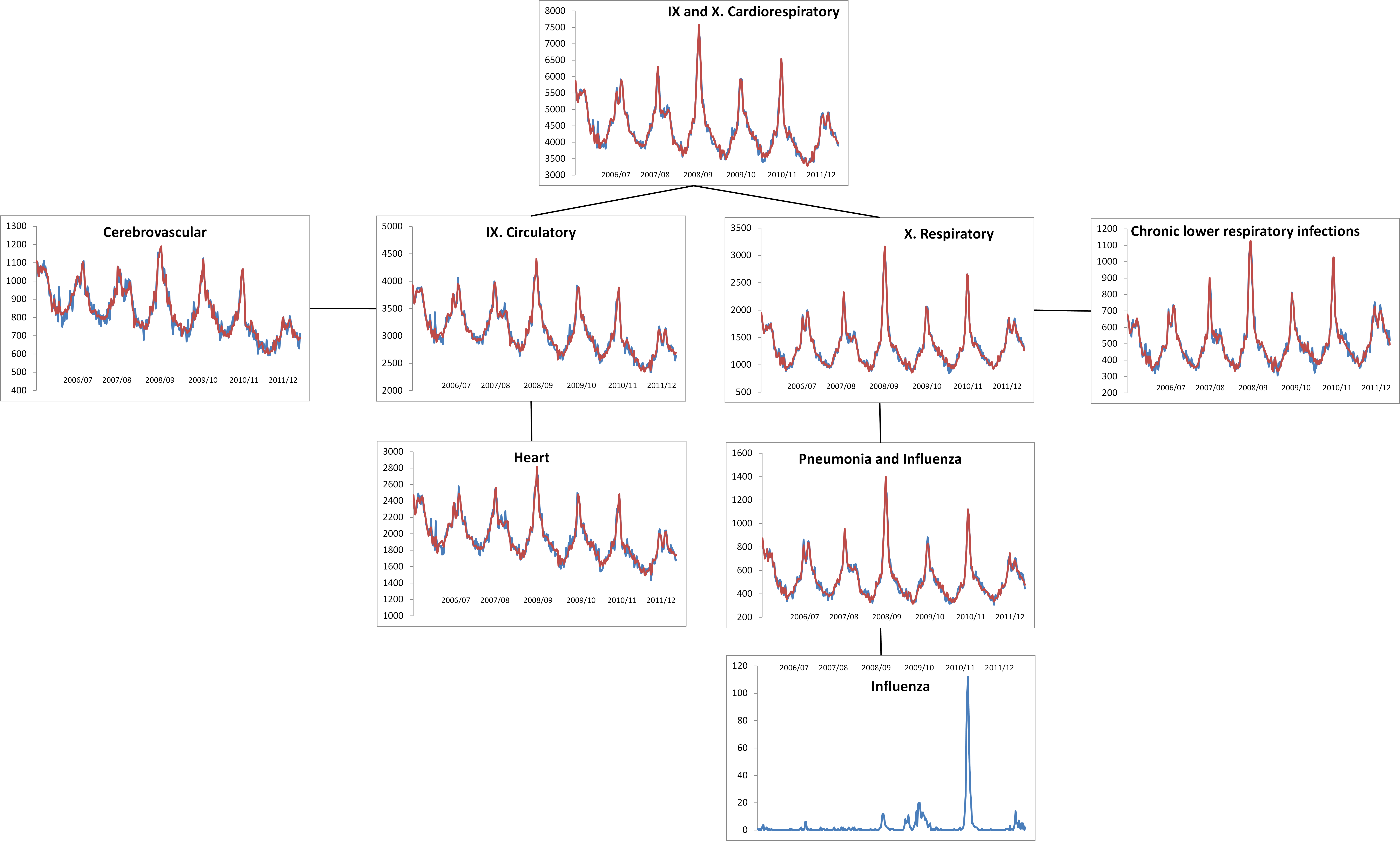

Supplement: Figure S1 — Observed number of all-age weekly deaths by primary cause of death (blue) and expected deaths from final model (red) for cardiorespiratory causes. (TIF) [file pone.0079360.s001.tif]

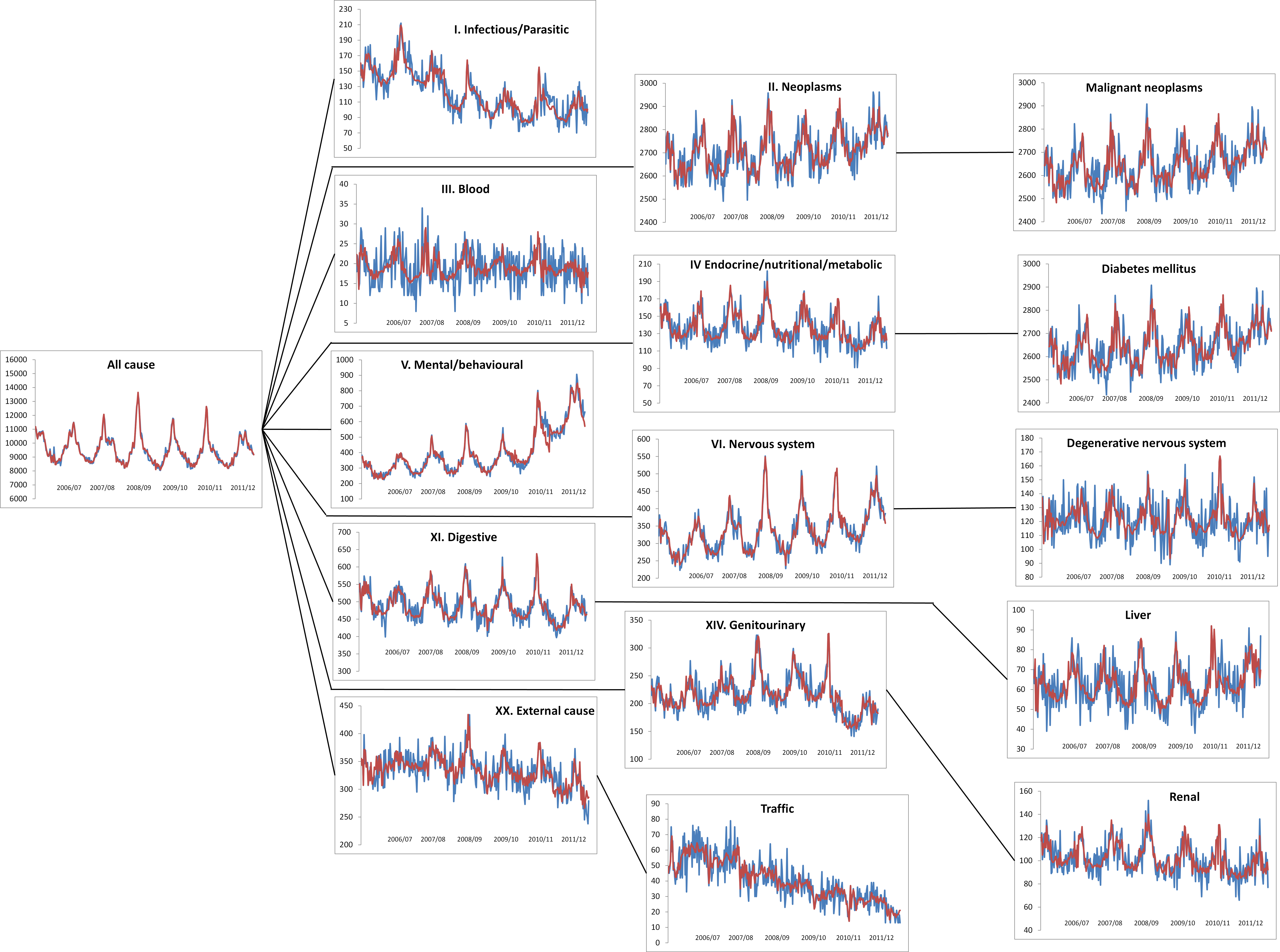

Supplement: Figure S2 — Observed number of all-age weekly deaths by primary cause of death (blue) and expected deaths from final model (red) for causes other than cardiorespiratory. (TIF) [file pone.0079360.s002.tif]

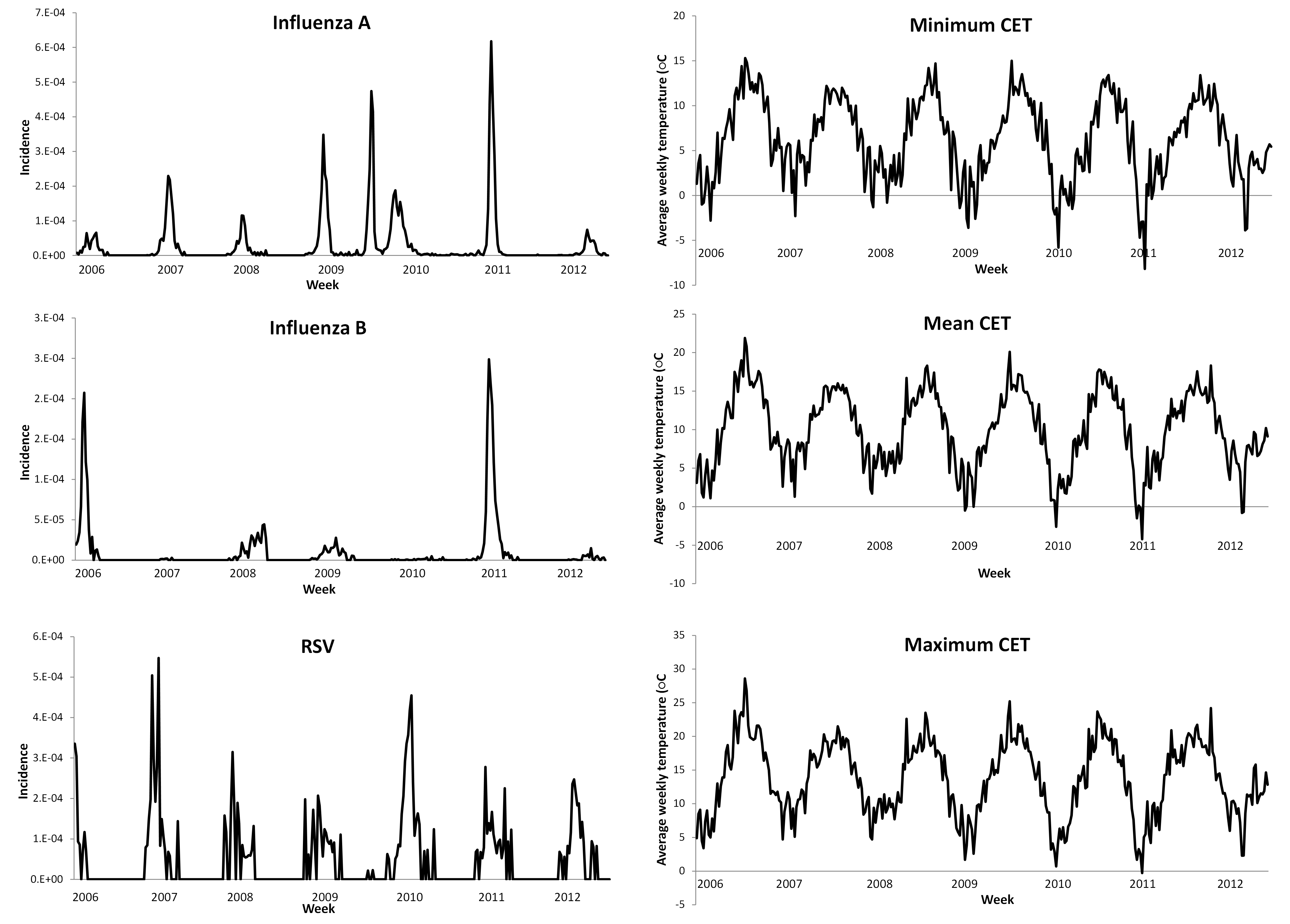

Supplement: Figure S3 — Weekly incidence proxy (clinical activity multiplied by proportion of samples positive) for influenza A, influenza B and respiratory syncytial virus (RSV) and weekly means of minimum, mean and maximum Central England Temperature (CET), 2006–2012. (TIF) [file pone.0079360.s003.tif]

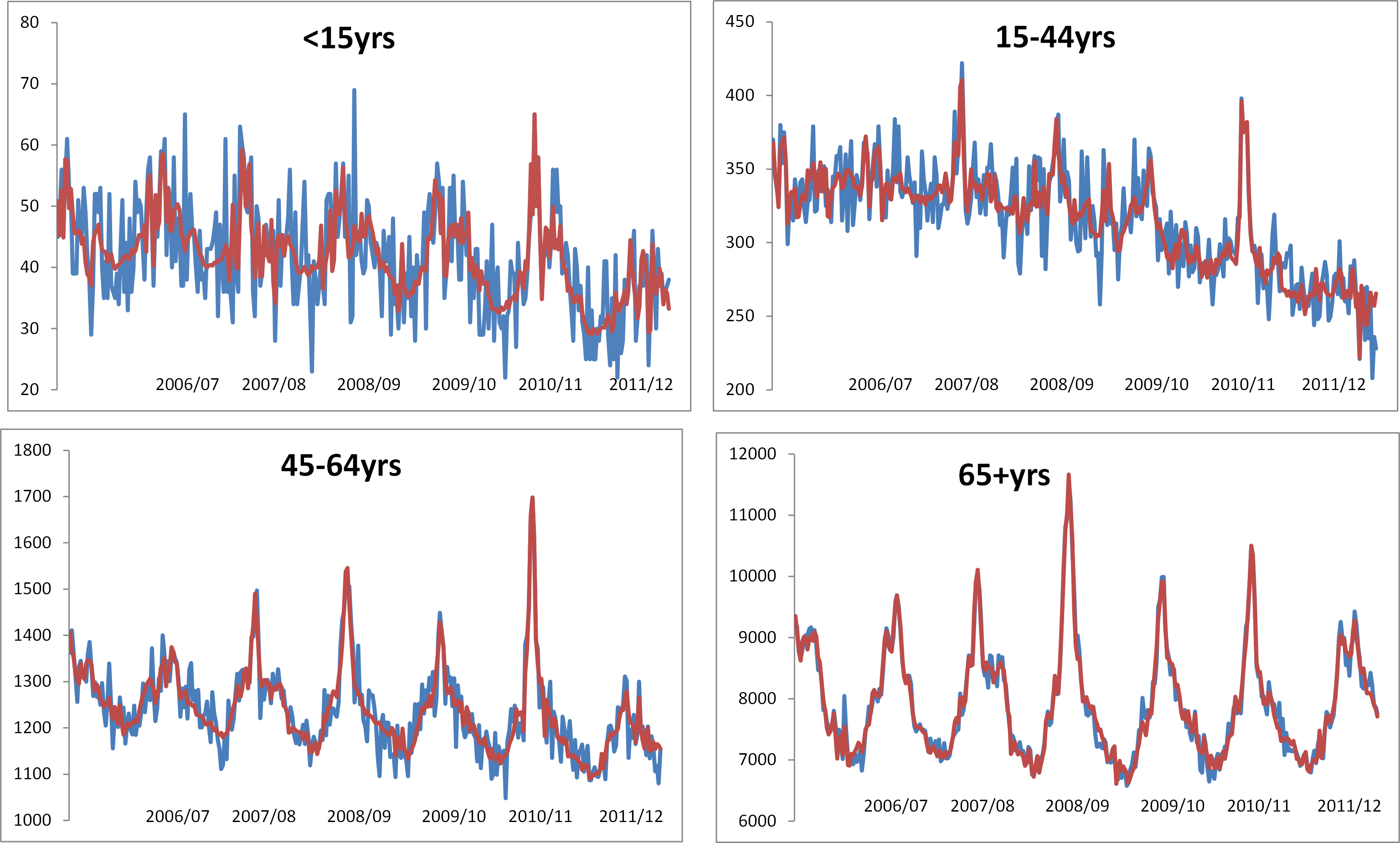

Supplement: Figure S4 — Observed number of weekly deaths by age group (blue) and expected deaths from final age-specific models (red) for all-cause deaths. (TIF) [file pone.0079360.s004.tif]

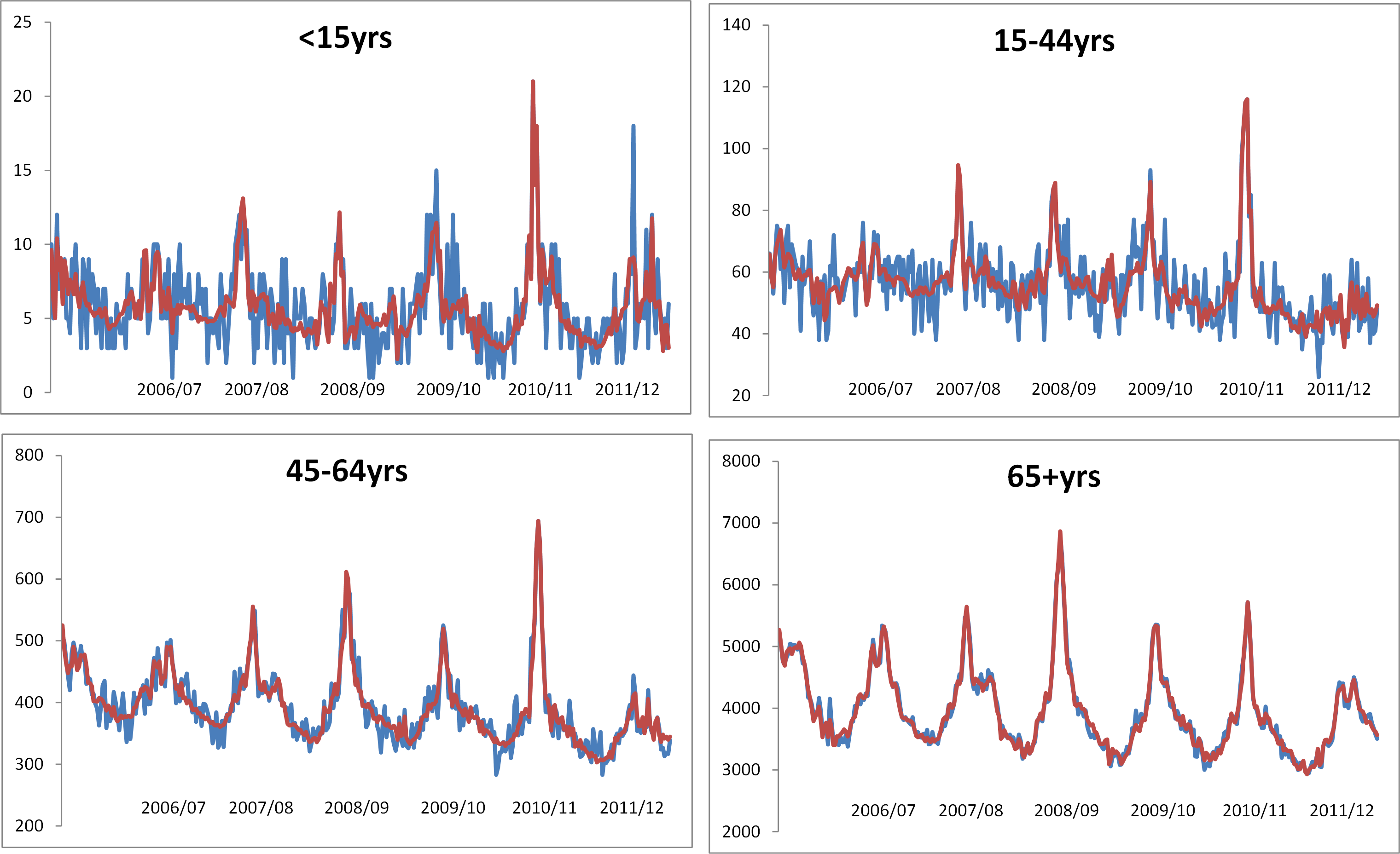

Supplement: Figure S5 — Observed number of weekly deaths by age group (blue) and expected deaths from final age-specific models (red) for cardiorespiratory deaths. (TIF) [file pone.0079360.s005.tif]

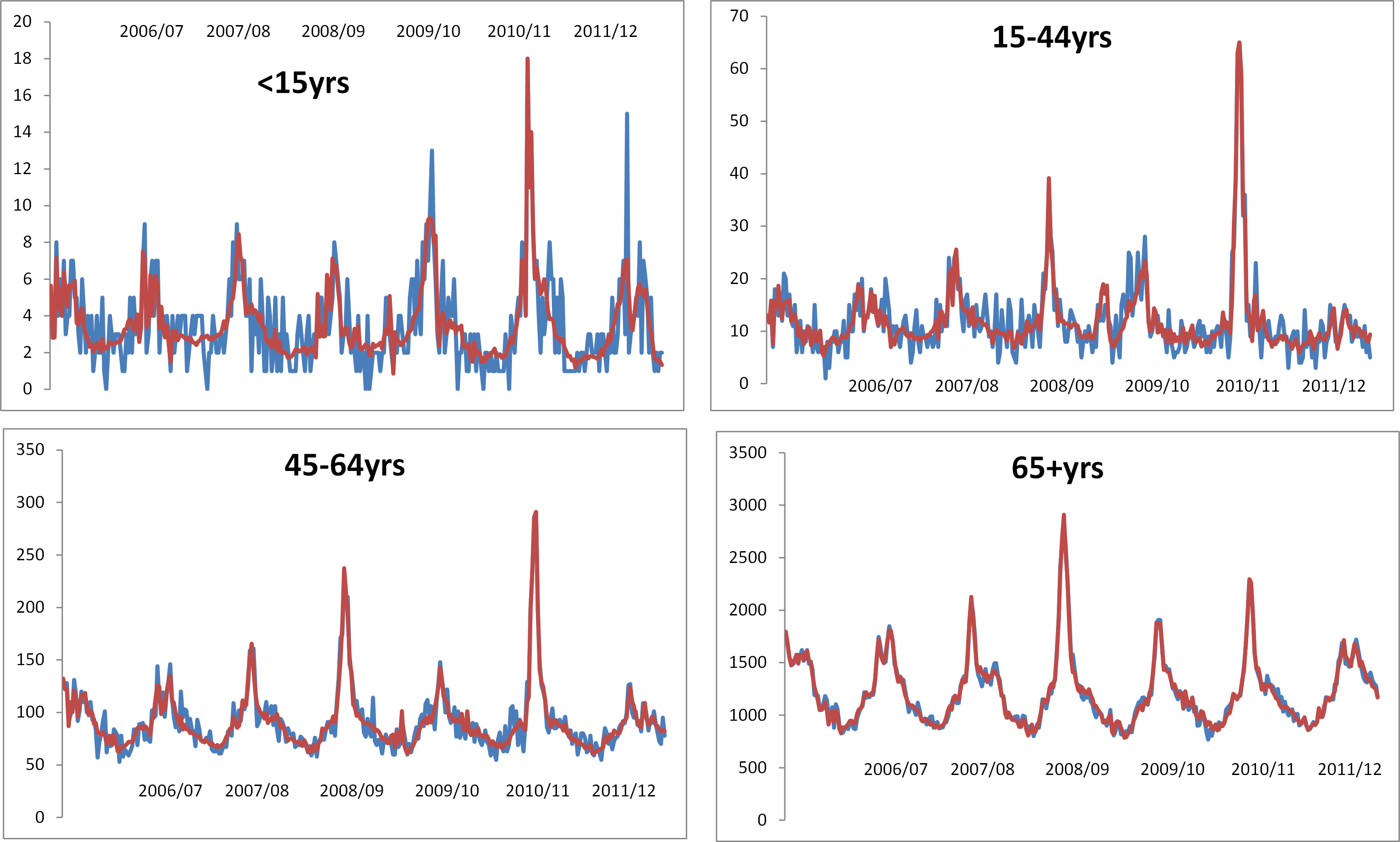

Supplement: Figure S6 — Observed number of weekly deaths by age group (blue) and expected deaths from final age-specific models (red) for respiratory deaths. (TIF) [file pone.0079360.s006.tif]

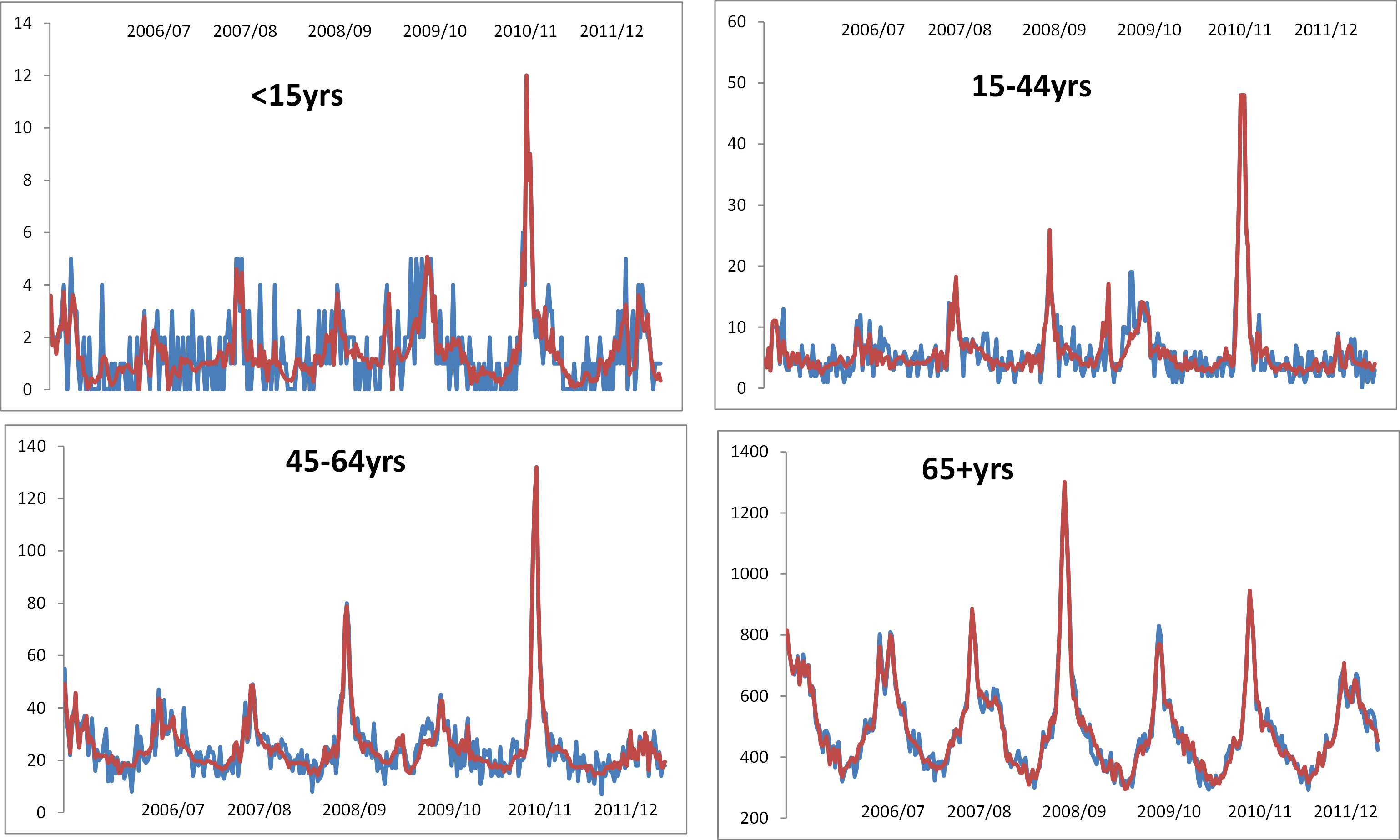

Supplement: Figure S7 — Observed number of weekly deaths by age group (blue) and expected deaths from final age-specific models (red) for pneumonia and influenza deaths. (TIF) [file pone.0079360.s007.tif]
