# Supplementary material for: Mortality Attributable to Influenza in England and Wales Prior to, during and after the 2009 Pandemic
Source: PLoS One. 2013 Dec 11;8(12):e79360. doi: 10.1371/journal.pone.0079360 (PMC3859479; doi:10.1371/journal.pone.0079360)
Supplement: Table S1 — List of ICD-10 codes extracted with description. (DOCX) [file pone.0079360.s008.docx]

| **Chapter** | **Description** | | | **Cause** | | | **Code** |
| --- | --- | --- | --- | --- | --- | --- | --- |
| I | Certain infectious and parasitic diseases | | | | | | A00-B99 |
| II | Neoplasms | | | | | | C00-D48 |
|  |  |  | | Malignant neoplasms | | | C00-C97 |
| III | Diseases of the blood and blood-forming organs | | | | | | D50-D89 |
| IV | Endocrine, nutritional and metabolic diseases | | | | | | E00-E90 |
|  |  |  | | Diabetes mellitus |  |  | E10-E14 |
| V | Mental/behavioural disorders | | | | | | F00-F99 |
| VI | Nervous system | | | | | | G00-G99 |
|  |  | | | Degenerative disorders | | | G30,G31,G32 |
| IX and X | Cardiorespiratory | | |  |  |  | I00-J99 |
|  |  | | IX. Circulatory |  |  |  | I00-I99 |
|  |  | |  | Cerebrovascular diseases | | | I60-I69 |
|  |  | |  | Heart diseases | | | I00-I09,I11,I13,I20-I51 |
|  |  | |  |  | Ischaemic heart diseases | | I20-I25 |
|  |  | |  |  |  | AMI^1^ | I21,I22 |
|  |  | | X. Respiratory | | | | J00-J99 |
|  |  | |  | Influenza and pneumonia | | | J09-J18 |
|  |  | |  | Chronic lower respiratory infections | | | J40-J47 |
| XI | Diseases of the digestive system | | |  |  |  | K00-K93 |
|  |  | | | Liver disease | | | K70,K73,K74 |
| XIV | Diseases of the genitourinary system | | |  |  |  | N00-N99 |
|  |  | | | Renal disease |  |  | N00-N07,N17-N19,N25-N27 |
| XX | External cause | | | | | | V01-Y98 |
|  |  | | | Traffic |  |  | V01-V99 |

^1^Acute Myocardial Infarction
